# Vestibular modulation of spatial perception

**DOI:** 10.3389/fnhum.2013.00660

**Published:** 2013-10-10

**Authors:** Elisa R. Ferrè, Matthew R. Longo, Federico Fiori, Patrick Haggard

**Affiliations:** ^1^Institute of Cognitive Neuroscience, University College LondonLondon, UK; ^2^Department of Psychological Sciences, Birkbeck University of LondonLondon, UK; ^3^Department of Brain and Behavioural Sciences, University of PaviaPavia, Italy

**Keywords:** galvanic vestibular stimulation, vestibular system, line bisection, space perception, unilateral spatial neglect

## Abstract

Vestibular inputs make a key contribution to the sense of one’s own spatial location. While the effects of vestibular stimulation on visuo-spatial processing in neurological patients have been extensively described, the normal contribution of vestibular inputs to spatial perception remains unclear. To address this issue, we used a line bisection task to investigate the effects of galvanic vestibular stimulation (GVS) on spatial perception, and on the transition between near and far space. Brief left-anodal and right-cathodal GVS or right-anodal and left-cathodal GVS were delivered. A sham stimulation condition was also included. Participants bisected lines of different lengths at six distances from the body using a laser pointer. Consistent with previous results, our data showed an overall shift in the bisection bias from left to right as viewing distance increased. This pattern suggests leftward bias in near space, and rightward bias in far space. GVS induced strong polarity dependent effects in spatial perception, broadly consistent with those previously reported in patients: left-anodal and right-cathodal GVS induced a leftward bisection bias, while right-anodal and left-cathodal GVS reversed this effect, and produced bisection bias toward the right side of the space. Interestingly, the effects of GVS were comparable in near and far space. We speculate that vestibular-induced biases in space perception may optimize gathering of information from different parts of the environment.

## Introduction

The sense of one’s own position, orientation and motion in three-dimensional space derives from the integration of a variety of signals, including muscles, joints, vision, touch, and vestibular inputs (Lackner and DiZio, 2005). The vestibular system contains two distinct structures: the semicircular canals, which detect changes in angular acceleration, and the otolith organs, which detect changes in linear acceleration and gravity. Both semicircular canals and otolith organs constantly provide information to the brain regarding our body’s position and movement. Thus, the vestibular signals are crucial to spatial perception (Villard et al., 2005; Clement et al., 2009, 2012).

Several studies focussed on the vestibular contribution to spatial perception in neurological patients. Patients with unilateral spatial neglect (USN) fail to detect objects or to perform movements in the space contralateral to the cerebral lesion. The classic lesion site is the parietal lobe of the right hemisphere (Vallar, 1998; Bisiach and Vallar, 2000; for review see Kerkhoff, 2001). Line bisection is one of the most common tests for assessing USN (Albert, 1973). Patients are instructed to visually examine a horizontal line, generally presented on a sheet of paper aligned with the patient’s trunk midline, and to indicate its center using a pencil. USN patients locate the bisection point shifted toward the ipsilesional side of the space, so that right hemisphere damaged patients produce a characteristic rightward error in bisection (Heilman and Valenstein, 1979; Schenkenberg et al., 1980; Milner et al., 1993; Doricchi and Angelelli, 1999; Daini et al., 2002).

Vestibular stimulation was one of the first sensory stimulations used in order to modulate USN (Silberfenning, 1941). Rubens (1985) applied cold caloric vestibular stimulation (CVS) to the auditory canal of the left ear in right brain-damaged patients. This transiently improved signs related to USN. More recently, Rorsman et al. (1999) reported a reduction of USN in a visuo-motor task (line cancellation task) during left-anodal and right-cathodal galvanic vestibular stimulation (GVS). Importantly, these findings suggest a stimulation effect beyond the oculo-motor vestibular reflex and the spontaneous recovery. Similarly, left-anodal and right-cathodal GVS ameliorates visuo-constructive deficits in the Rey figure (Wilkinson et al., 2010) and the rightward bias in the bisection task (Utz et al., 2011). The recovery of USN (Cappa et al., 1987; Bisiach et al., 1991; Rode and Perenin, 1994), and the demonstration of contralateral cortical activation after vestibular stimulation (Fink et al., 2003) suggested an interaction between vestibular stimulation and spatial perception: amelioration of USN may depend on the activation of cortical areas receiving vestibular projections in the right hemisphere.

In contrast to the clear effects in patients, the contribution of vestibular inputs to space perception in normal cognition remains unclear. On the one hand, vestibular stimulation might act on non-specific mechanisms, such as general attention or arousal. On the other hand, vestibular inputs might directly affect spatial processing. Several previous studies investigated low-level visuo-vestibular mechanisms for orienting the gaze (Angelaki and Cullen, 2008), or perceiving the subjective visual vertical (Bohmer and Mast, 1999). Rorden et al. (2001) found no shifts of visuo-spatial attention following CVS in a Posner-like task (Posner, 1980). In contrast, natural vestibular activation induced by passive whole-body rotation influenced the allocation of spatial attention toward the side of rotation (Figliozzi et al., 2005). However, this form of vestibular stimulation will inevitably also activate other afferents, including those from cutaneous and proprioceptive receptors. Thus, differences between the types of vestibular stimulation used and the consequent activations of vestibular and other afferents might explain the contrasting findings (Lopez et al., 2012). No previous study has demonstrated a laterality-specific shift of spatial representation in healthy participants using purely vestibular stimulation.

In the present study, therefore, we examined whether vestibular stimulation alters the perception of position along the left-right spatial dimension. Further, we investigated whether vestibular stimulation also influences the transition between near and far space, i.e., depth or 3D space. We adapted Longo and Lourenco’s (2006) paradigm, in which participants bisected lines located at several distances using a laser pointer. In standard paper-and-pencil line bisection tasks, healthy participants generally mis-bisect horizontal lines slightly to the left, a phenomenon known as “*pseudoneglect*” (Bradshaw et al., 1987; Manning et al., 1990; Chokron and Imbert, 1993; McCourt and Jewell, 1999; Jewell and McCourt, 2000). A number of studies have demonstrated that the leftward bias in near space shifts gradually with increased viewing distance to become a rightward bias in far space (e.g., McCourt and Garlinghouse, 2000; Varnava et al., 2002; Longo and Lourenco, 2006, 2007; Gamberini et al., 2008; Lourenco and Longo, 2009). This rightward transition occurs between distances within arm’s reach, outside of arm’s reach, as well as distances crossing this boundary, suggesting that there is no discrete border of near space (Longo and Lourenco, 2006). Nevertheless, the *rate* at which the transition occurs is correlated both with arm length (Longo and Lourenco, 2007) and with self-reported claustrophobic fear (Lourenco et al., 2011), suggesting that the “*size*” of near space can be quantified in terms of how rapidly bisection biases change with viewing distance.

We delivered binaural GVS to non-invasively activate the vestibular organs (i.e., both otoliths and semicircular canal afferents, Stephan et al., 2005). An anode and cathode were placed on the left and right mastoid, or vice versa. Perilymphatic anodal currents hyperpolarize the trigger site and lead to inhibition, whereas cathodal currents depolarize it resulting in excitation (Goldberg et al., 1984). This induces a polarity-dependent “*virtual rotation vector*” (Day and Fitzpatrick, 2005) which can influence orientation perception and posture. More surprisingly, GVS also causes polarity-dependent modulation of sensory and cognitive functions (see Utz et al., 2010 for a review). These behavioral effects are consistent with neuroimaging evidence revealing asymmetrical cortical vestibular projections in the non-dominant hemisphere (Dieterich et al., 2003). Here we hypothesized that left-right spatial perception would be affected by GVS: we predicted that left-anodal and right-cathodal GVS would induce a leftward bias in the line bisection by activating the right hemisphere. In contrast, the opposite polarity of GVS, i.e., right-anodal and left-cathodal, would induce a rightward bias by activating the left hemisphere. An additional point of interest would be any interaction between vestibular stimulation and viewing distance—such as a difference between the effects of GVS on bisection in near compared to far space.

## Materials and methods

### Participants

Fourteen naïve right-handed paid participants volunteered in the study (9 male, ages mean ± SD: 26.7 ± 4.19 years). Participants with a history of visual, vestibular or auditory disorders were excluded. Informed consent was obtained prior to participation in the experiment. The experimental protocol was approved by University College London research ethics committee.

### Galvanic vestibular stimulation

**Figure 1:**
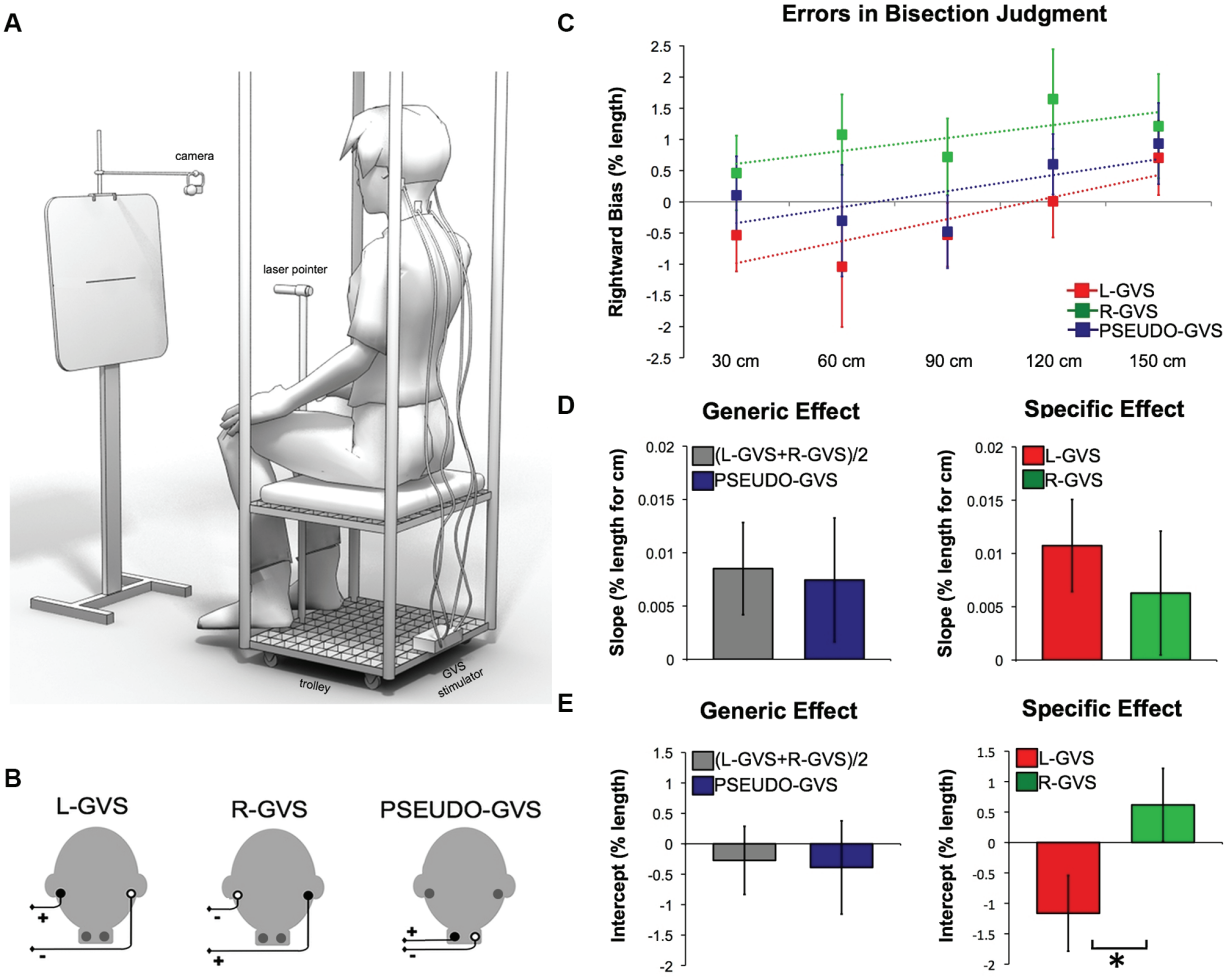
**Effects of GVS on spatial perception.** **(A)** Experimental set-up. **(B)** GVS polarities and electrodes configurations. **(C)** Errors in bisection judgment. Raw data in each condition in function of the distances and fitted linear regression (dashed lines). **(D)** Slope data as a function of GVS condition. **(E)** Intercept data as a function of GVS condition.

Bipolar GVS was used to deliver a boxcar pulse of 1 mA with 8 s of duration, via a commercial stimulator (Good Vibrations Engineering Ltd., Nobleton, Ontario, Canada). Carbon rubber electrodes (area 10 cm^2^) were placed binaurally over the mastoid processes and fixed in place with adhesive tape. The areas of application were first cleaned with cotton wool soaked in surgical spirit, and electrode gel was applied to reduce the impedance. The left-anodal and right-cathodal configuration is named “L-GVS” following previous convention (Ferrè et al., 2013a,b). The inverse polarity, namely left-cathodal and right-anodal configuration, is named “R-GVS” (Figure 1B). This GVS configuration induces sensations of head movement, illusory perception of motion and it evokes postural movements in the direction of the anodal ear (Day et al., 1997). A skin tingling sensation is reported to be stronger on the cathodal side. Importantly, no long-lasting effects have been described delivering low intensity (1 mA) and short duration (8 s) bipolar GVS. A sham stimulation, “PSEUDO-GVS”, based on that used by Lopez et al. (2010), was applied attaching the electrodes on the left and right side of the neck, about 5 cm below the GVS electrodes, with left anodal and right cathodal configuration (Figure 1B). This causes a similar tingling skin sensation to real GVS but without stimulating the vestibular organs. It functions as a control for non-specific alerting effects. In our experiment, such non-vestibular effects could include skin sensations generated by the GVS electrodes, and also the knowledge that an unusual stimulation is occurring.

### Stimuli and procedure

Verbal and written instructions about the task were given to participants at the beginning of the session. Participants performed a line bisection task during L-GVS, R-GVS or PSEUDO-GVS. Electrodes for GVS and PSEUDO-GVS were placed at the beginning of the session and remained in place for the entire duration of the experiment (Figure 1B). The electrodes and the polarity of stimulation were selected under randomized computer control.

To reduce the postural consequences of vestibular input, the experiment was conducted in a comfortable sitting position. This also reduced the tendency to tilt towards the anodal side during GVS. Participants were seated on a movable custom-built trolley whose seat was 71 cm above the floor. They were required to bisect lines presented at different distances in space (Figure 1A). The participant’s head was in a neutral posture, i.e., neither tilted nor flexed, for all the duration of the task. A laser pointer was attached to the head of a tripod fixed to the trolley 117 cm above the floor (Figure 1A). Participants used their right hand to adjust the left-right position of the tripod head, so as to bisect the line with the laser beam. Participants were instructed to move the beam downwards to the floor on the left or the right side at random, after each bisection.

A panel holding the stimuli was placed in front of the participant (Figure 1A). A camera (Logitech Webcam Pro 9000) was suspended on a tripod directly above the center of the panel and aligned with the lines. The camera was controlled by a computer custom-build program that captured JPEG images (1280 × 960 pixels) of the panel, including the line and the bisecting laser beam, and saved them for off-line coding. Stimuli consisted of lines of 10, 20 and 30 cm (1 mm in height) centered on 29.7 cm × 42 cm sheets of paper attached horizontally to the panel. Each line was presented 115.5 cm above the floor. The distance from the participant to the line could be 30 cm, 60 cm, 90 cm, 120 cm or 150 cm. These distances were controlled by an experimenter pushing the trolley on which the participant was seated to the appropriate location, marked on the floor with tape. As line length was held constant across distances, angular size varied with increasing distance.

A total of ten blocks were administered, two for each distance from the participant to the line. The order of blocks was randomized across participants. Each block comprised nine trials in random order, defined by factorial combination of line length (10 cm, 20 cm and 30 cm) and stimulation (L-GVS, R-GVS and PSEUDO-GVS). On each trial, L-GVS, R-GVS or PSEUDO-GVS was delivered after 1 s from the beginning of the trial. Then, after 1 s, a tone signalled participants to open their eyes, to point to the center of the line with the laser beam, and maintain the pointing location until a further tone occurred 6 s later. The images were captured during the interval of time between the two tones. Participants were instructed to move the beam to the center of the line, and then hold it there without making further adjustments. This instruction was designed to prevent participants from exploring the space.

### Data analysis

The pixel coordinates of each pointing laser beam, and each left and right extreme of the line were measured on each image using ImageJ software.^1^ Errors in bisection were calculated as mean rightward deviations from the objective center of the line expressed as a percentage of line length, and were calculated for each participant for each distance (30 cm, 60 cm, 90 cm, 120 cm, 150 cm) in each experimental condition (L-GVS, R-GVS, PSEUDO-GVS).

We fitted a linear regression to model bisection error as a function of distance for each condition and for each participant. Line bisection bias has been considered as the combination of two factors (Longo and Lourenco, 2006). First, the slope of the relation between bisection error and distance is a measure of the “*size*” of near space, reflecting the rate at which the bisection bias shifts rightward with increasing distance. Second, the intercept of the fitted lines represents the leftward/rightward bias at hypothetical distance zero, and thus the general lateral shift of spatial representation. Slope and intercept are logically independent (Longo and Lourenco, 2006): experimental manipulations can induce a reduction/increase of slope without a corresponding change in intercept or vice versa. Accordingly, estimates of slope and y-intercept were used for subsequent analyses.

Slope and intercept values were compared across different stimulation conditions using planned contrasts. We hypothesized that vestibular stimulation might influence the slope and intercept in two distinct ways (Ferrè et al., 2013a,b). First, any activation of the vestibular system might influence bisection independent of polarity and hemispheric effects, perhaps because of generic effects such as general arousal. To test this *generic hypothesis*, we compared the average of the L-GVS and R-GVS conditions to the PSEUDO-GVS condition, for each dependent variable (slope and intercept). Second, we hypothesized that the effects of vestibular stimulation could be *specific* to the hemisphere activated, and would therefore differ between L-GVS and R-GVS conditions. Our planned contrasts thus reflect hypothesis about plausible ways that vestibular stimulation might influence spatial perception. Distinguishing generic and specific effects of an intervention is an established method in biosciences, and has been used previously for vestibular interventions (Schmidt et al., 2013).

## Results

Analysis of regression slopes showed a systematic shift in the bisection bias toward the right with increasing distance. This bias was found across all conditions (*t*_(13)_ = 2.068, *p* = 0.05) and it is consistent with previous results (Varnava et al., 2002; Longo and Lourenco, 2006, 2007; Gamberini et al., 2008; Lourenco and Longo, 2009; see data in Figure 1C).

To identify whether *generic* vestibular input influences the perception of space, we compared (L-GVS + R-GVS)/2 to PSEUDO-GVS condition. This planned comparison revealed no significant difference in slope values reflecting the transition between near and far space (*t*_(13)_ = 0.264, *p* = 0.796). Similarly, intercept values representing the leftward/rightward bias were not different between conditions (*t*_(13)_ = 0.301, *p* = 0.768, Figure 1D).

To investigate the *specific* vestibular effect, we directly compared L-GVS to R-GVS conditions. This contrast was designed to reveal how vestibular projections in each hemisphere might influence the cognitive processes involved in space perception. No significant differences were found in slope values (*t*_(13)_ = 0.686, *p* = 0.505). In contrast, intercept values revealed a significant difference between L-GVS and R-GVS (*t*_(13)_ = −3.613, *p* = 0.003, Figure 1E). L-GVS induced a bias toward the left side of the space, while R-GVS toward the right.

## Discussion

Information from the vestibular peripheral organs in the inner ear is integrated with several other classes of signals about the body. Low-level interactions between vestibular and visuo-spatial information are essential in providing the organism with space representation (Villard et al., 2005; Clement et al., 2009, 2012). However, the vestibular contribution to perceiving environmental space has proved difficult to study. Here, we demonstrated that vestibular input *in general* did not influence spatial processing: neither horizontal left/right spatial representation, nor the transition between near and far space. In contrast, *polarity-specific* vestibular input had differential effects on spatial perception, broadly consistent with those previously reported in USN patients: left-anodal and right-cathodal GVS, which is considered to activate the vestibular projections in the right hemisphere, induced a leftward bisection bias, while right-anodal and left-cathodal GVS produced a bias towards the right side of space. These left-right biases caused by GVS were comparable in near and far space.

GVS polarity-dependent differences in postural, sensorimotor and cognitive functions have previously been demonstrated both in healthy volunteers and in brain damaged patients (Utz et al., 2010). L-GVS decreases the firing rate of the vestibular nerve on the left side and increases it on the right side (Goldberg et al., 1984; Day and Fitzpatrick, 2005), while R-GVS has the opposite effect. Neuroimaging studies have revealed asymmetrical cortical vestibular projections, suggesting that the core region of the vestibular network is primarily located in the non-dominant right hemisphere in right-handed subjects (Bense et al., 2001; Suzuki et al., 2001; Dieterich et al., 2003; Janzen et al., 2008). Additionally, Fink et al. (2003) used fMRI to study the effects of bipolar GVS. They found that left-anodal and right-cathodal L-GVS produced unilateral activation of the right hemisphere vestibular projections, while the opposite polarity, i.e., left-cathodal and right-anodal GVS, activated both left and right hemispheres (Fink et al., 2003).

Two alternative mechanisms could explain our results. First, R-GVS and L-GVS might diffusely activate a large-scale hemispheric network for spatial attention. The activation of each cerebral hemisphere would lead to a contralateral attentional bias (Kinsbourne, 1987; Làdavas et al., 1989; Corbetta et al., 1995) as observed in our study. Alternatively, vestibular input might project to specific cortical areas within each hemisphere involved in spatial processing. Vestibular inputs have not been found to project to any *primary* cortical area. Rather neuroimaging studies identified a network of activations induced by vestibular stimulation, involving the posterior and anterior insula, the temporoparietal junction, the inferior parietal lobule, the somatosensory cortices, the primary motor cortex and premotor cortex (Bottini et al., 1994; Bense et al., 2001; Fasold et al., 2002; Emri et al., 2003). We speculate that the leftward bias on bisection tasks caused by L-GVS would result from an over-excitation caused by vestibular stimulation of the right posterior parietal cortex. Conversely, the rightward bias induced by R-GVS could reflect the activation of the homologous areas in the left hemisphere. However, it remains unclear if our results reflect activations which produce a diffuse imbalance *between* hemispheres, or whether specific activations *within* each hemisphere are responsible.

Recent clinical studies in patients with peripheral vestibular disorders support the hypothesis of spatial representation changes based on vestibular induced changes. Saj et al. (2013) described severe horizontal deviations in the representation of body orientation after unilateral vestibular loss. Interestingly, they found that only patients affected by left peripheral vestibular loss showed changes in perception of body orientation in space, suggesting not only a role of the vestibular system in the processing of space, but also a right hemispheric dominance.

Could the bias in bisection be a non-specific effect of GVS? For example, GVS influences balance and postural control, producing compensatory postural sway in the direction of the anode (Day and Fitzpatrick, 2005). However, an indirect effect on bisection by postural responses seems unlikely. The effects induced by GVS on postural responses have been mainly demonstrated in standing participants, while participants in our study were seated. Importantly, GVS was delivered not at the beginning of bisection response, but several seconds before. Therefore, any postural adjustments should have been stabilized at the time of the bisection task. GVS also induced a tingling skin sensation that could have acted as a cue for the participant. If so, GVS might change general arousal, or drive attention toward the cathodal side, where the tingling sensation is reported to be strongest. This would not explain the shift induced toward the anodal side in both GVS conditions observed in our data. Thus, our results do not imply a general non-specific bias in bisecting, but rather a high-level spatial modulation.

In conclusion, our results showed that vestibular inputs shift spatial attention towards one side of the horizontal space, as result of the activation of the vestibular projections in the contralateral hemisphere. Every movement of the head implies a new relation between the organism and the surrounding space to acquire salient information from the environment. We suggest the vestibular organs may optimize gathering of information from different parts of the environment.

## Conflict of interest statement

The authors declare that the research was conducted in the absence of any commercial or financial relationships that could be construed as a potential conflict of interest.

## Footnotes

^1^http://rsbweb.nih.gov/ij/
